# Editorial: Exploring the role of T helper cells in autoimmune disease

**DOI:** 10.3389/fimmu.2024.1434684

**Published:** 2024-06-04

**Authors:** Dariush Haghmorad, Valentyn Oksenych, Peter C. Huszthy

**Affiliations:** ^1^ Department of Immunology, School of Medicine, Semnan University of Medical Sciences, Semnan, Iran; ^2^ Broegelmann Research Laboratory, Department of Clinical Science, University of Bergen, Bergen, Norway; ^3^ Department of Microbiology and Infection Control, Akershus University Hospital, Lørenskog, Norway; ^4^ Institute of Clinical Medicine, University of Oslo, Oslo, Norway

**Keywords:** autoimmune disease, T peripheral helper cells, T follicular helper cells, T regulatory cells, B cells, autoantibodies

Autoimmune diseases (AD) are on the rise (1), currently affecting one in ten according to a recent population-based study (2). With more than 100 recognized distinct clinical entities, AD have complex aetiologies and a biological diversity that does not easily lend itself to simplistic treatment strategies. Although B cell depletion is an applied clinical treatment form for AD, there are several drawbacks to this therapy (3). Yet another logical point of attack is to target autoreactive CD4^+^ T helper (Th) cells. Th cells that reside in B cell follicles (the Tfh subset) play a major role in promoting the production of high-affinity autoantibodies (4). In conventional T-B collaboration, the Tfh are thought to share the B cell’s specificity for a given auto-antigen. In the more complex case of idiotype (Id) driven T-B collaboration, Tfh are specific for BCR V-region peptides presented on MHCII molecules on the B cell’s surface (5). Id-driven T-B collaboration may underlie AD, as demonstrated in mouse models (6).

To control AD through T cell-targeted therapies, e.g. through skewing disease-promoting effector cells (Th subsets) toward a regulatory (Treg) phenotype (7, 8), we need to comprehend more of the T cells’ biology in these disorders. In an effort to contribute to this understanding, the current Research Topic covers recent advances that underlie the pathological Th/Treg cell imbalance in a variety of AD. More specifically, the authors uncover the roles and activation mechanisms of pathogenic peripheral T helper (Tph) cells as well as mechanisms whereby tissue-resident Tregs are attenuated through truncation or transcriptional silencing of the main regulator, *Foxp3*.

Julé et al. (**Table 1**) have immunophenotyped the dominant T- and B cell compartment in juvenile oligoarthritis (JIA), a distinct entity of chronic arthritis unique to children. In JIA, antinuclear Ab (ANA) dominate, whereas anti-rheumatoid-factor antibodies typical of RA are missing. In ANA^+^ JIA, lymphocytic (i.e. T cell-B cell) aggregates and plasma cell infiltrations are present in the joint. The authors found that in the synovial fluid of ANA^+^ JIA patients, a highly activated and Tph subset dominates characterized by genes associated with T cell help to B cells. Alongside Tph-like Tregs were recovered. Tph-like Tregs are PD-1^hi^ and express CXCL13 and ICOS. Tregs did not differ in frequency between ANA^+^ and ANA^-^ JIA patients. Thereby, the authors suggest that a predominance of B cell helping pathogenic Tph rather than a loss in attenuating Tph-like Treg contributes to ANA production and subsequent disease. The *ID2* and *BHLHE40* genes are upregulated in JIA Tph and Tph-like Treg; the transcriptional programs promoted by these regulators may serve to retain both these T cell subsets at this extralymphoid anatomical site.

**Table 1:** Summary of the research articles and reviews published in the Research Topic.

Article (first author)	Article type	Disease entity studied or reviewed	Impacted organ(s)	Mode of tissue destruction	Auto-Ab targets	T cell imbalance	Cytokines and chemokines	Associated genetic factors or signaling pathways discussed
Mazzieri et al.	Mini-review	Hashimoto’s thyroiditis (HT)	Thyroid	APC→CD4^+^ Th, CD8^+^ CTL, cytokines→TD, fibrosis	TPO, Tg	Th1↑Th17↑γ Treg loss of function	IL-17↑, IL-22↑	*HLA*-polymorphisms, *FOXP3*Δ2, *BACH2*
Julé et al.	Research paper	Juvenile oligoarthritis (JIA)	Joints	APC→CD4^+^ Th, B cells, MΦ→proteases, INF mediators, Auto-Ab→TD	Nuclear Ag	Th1↑ Th17↑γ Treg loss of function	IL-21↑, TH17→IL6, MMP1, MMP3,IL8	*ID2*, *BHLHE40*, *ALOX5AP*
Garthsteyn et al.	Research paper	Lupus nephritis (SLE)	Kidney	APC→CD4^+^ Th cells→B cells/Tfh, Tph→Plasma cells→Auto-Ab→immune complex formation→CA, INF→TD	Nuclear Ag	Th1↑,Th2↓, Th17↑, Treg↓	IL-21↑	↑SAP, ↑TCR signaling, ↑NF-κB signaling, SLAM signaling, chemokine signaling, Toll-like receptor signaling
Horellou et al.	Research paper	MOGAD	CNS	Peripheral trigger→MOG-sp. B cell + Tfh→MOG-sp. PC and CD4^+^ Th→CNS entry→TD	Myelin oligodendrocyte glycoprotein (MOG)	Th2↑ Th17↑; Treg↓ in relapsing patients	↑IL-6, IL-17, G-CSF, TNF-α,BAFF, APRIL, CXCL13, CCL19	↓Foxp3 mRNA, methylation of *CNS2* (*FOXP3* locus)
Kalim et al.	Research paper	Fatal multiorgan inflammatory disorder (mouse model)	Colon, kidney, lung, liver	APC→CD4^+^ Th, CD8^+^ CTL→TD	not analyzed	Th1↑,Th2↑, Th17↑; Treg↓ (via apoptosis)	↑IFN-γ, IL-4, IL-17	↓Foxp3, ↑IFN-γ, ↑T-bet expression in the case of multiorgan inflammation
Sanders et al.	Review	Solid organ transplantation (SOT)	Kidney, liver, heart, pancreatic islets	1) APC (donor or host)→ CD4^+^ Th, B, Allo-Ab→TD; 2) peptide:MHCI (donor)→DC (host)→CD8^+^ CTL (semidirect recognition)	donor HLA (in chronic rejection)	Th1↑,Th2↑, Th17↑γ Treg↓	↑IL-2, IL-10, IL-35, IL-7, IL-15, IL-6	CpG-region demethylation (gene activation): *Foxp3, CD25*, *Ctla4*, *Tnfrsf18*, *Ikzf2, Ikzf4* (needed for Treg development)
Qi et al.	Review	Systemic AD: RA, SLE, SS; Organ-specific AD: MS, T1D	Several for SAD; CNS (for MS) and pancreas (for T1D)	several modes according to the given AD in question	several organ-specific or other targets	↑cTfh types 1–17, ↑GC Tfh; cTfr, GC Tfr ↓	↑IFN-γ (cTfh1); IL-4, IL-5, IL-13 (cTfh2); IL-17,IL-22 (cTfh17), IL-21 (Tfh)	ICOS→Bcl6 (Tfh, Tfr), ↓Bcl6 (cTfh), ↓ICOS (cTfr)
Manolios et al.	Review	T cell ion channels in SLE and other non-AD conditions like immunodeficiency and cancer	Kidney (SLE)	SLE: ↑CD39↑ATP→Inflammasome, IL-1β→TD	not discussed	Treg→Th17 conversion, ↓Treg, ↑Tfh (SLE)	↑IL-6, ↑IL-1β	Ca^2+^ release (SOCE), *P2RX7* (rs3751143) polymorphism; for SLE reduced response to ATP and a defect in apoptosis in Tfh

TPO, thyroperoxidase; Tg, thyroglobulin; TD, tissue destruction; CTL, cytotoxic T lymphocytes; MΦ, macrophages; APC, antigen-presenting cells; SAP, SLAM-associated protein; Ag, antigens; PC, Plasma cells; MOGAD, Myelin oligodendrocyte glycoprotein antibody-associated disease; CNS, central nervous system; CNS2, conserved non-coding sequence 2; CA, complement activation; INF, inflammation; Tfh, T follicular helper cell; SAD, systemic autoimmune diseases; cTFh, circulating Tfh; GC Tfh, germinal center Tfh; Tfr, T follicular regulatory cell; cTFr, circulating Tfr; RA, rheumatoid arthritis; SLE, systemic lupus erythematosus; GPA, granulomatosis with polyangiitis; SS, Sjögren’s syndrome; MS, multiple sclerosis; T1D, type 1 diabetes; P2RX7, Purinergic receptor P2X7; SOCE, store-operated Ca^2+^ entry.

↑, increased levels of; ↓, reduced levels of.

Mazzieri et al. (**Table 1**) have briefly reviewed some aspects concerning the immunological profile and the genetic susceptibility (HLA haplotypes) of Hashimoto’s thyroiditis (HT). The authors underline that it may be a functional deficiency rather than a numerical reduction in Tregs that contribute to HT pathogenesis. The authors discuss the propensity of Tregs to differentiate into Th types 1 and 2 following extrinsic signals as well as other ways Foxp3 levels may be attenuated to shift the balance away from suppression.

Gartshteyn et al. (**Table 1**) focused on peripheral T cells in systemic lupus erythematosus (SLE) patients with lupus nephritis. The signaling lymphocytic activation molecules (SLAM) are protein receptors on B- and T cell surfaces. SLAM-associated protein (SAP) functions in Tfh and Tph cells that are pathogenic CD4^+^ Th cell sub-populations in SLE. The authors hypothesized that Tph may underlie lupus nephritis pathogenesis. Lymphocytes from 30 SLE patients were included in the study; those with renal disease did indeed possess higher levels of SAP-positive Tph. The authors concluded that a better understanding of SAP and SLAM signaling might be instrumental in developing better therapeutic targets for patients with SLE combined with lupus nephritis.

Qi et al. (**Table 1**) focus on the roles of Tfh and T follicular regulatory cells (Tfr) in autoimmune diseases. As noted above, Tfh cells are necessary for promoting high-affinity antibody production by plasma cells (4), while Tfr cells suppress Tfh and B-lymphocytes, thus regulating the development and severity of autoimmune diseases. The authors review potential and known roles of Tfh and Tfr in autoimmune diseases, such as rheumatoid arthritis, SLE, Sjögren’s syndrome, granulomatosis with polyangiitis, type 1 diabetes, and multiple sclerosis. Moreover, the authors propose potential novel therapeutic strategies based on the regulation of Tfh and Tfr cells.

Manolios et al. (**Table 1**) focus on the roles of ion channels in T cell function and the pathologies associated with their dysfunction. Their summary includes channels for several ions (i.e. H^+^, Ca^2+^, K^+^, Na^+^, Mg^2+^, Zn^2+^, and Cl^-^). The authors conclude that plasma membrane-associated ion channels are relevant for drug development because they are targeted by approximately 10% of the marketed drugs.

Horellou et al. (**Table 1**) studied pediatric acquired demyelinating syndromes (ADS), focusing on MOG antibody-associated disease (MOGAD). They found that MOGAD patients had increased CD4^+^ Th2 and Th17 cells upon rh-MOG stimulation, indicating their role in MOGAD pathogenesis. CD4^+^ Th1 cell increase was significant in MOGAD but not in MS patients. Tregs responded differently based on disease subtype, with MOGNR patients showing increased CD4^+^Foxp3^+^ Tregs and MOGR patients showing decreased CD45RA^-^Foxp3^+^ Tregs. MOGR patients had a shift in Treg subpopulations, possibly contributing to disease progression. These findings highlight the importance of MOG-specific CD4^+^ T cells and Treg responses in MOGAD heterogeneity, suggesting potential therapeutic targets.

Kalim et al. (**Table 1**) revealed the crucial role of RhoA GTPase in Treg cells, influencing Treg cell homeostasis, autoimmune regulation, and tumor immunity. Mice lacking RhoA in Treg cells showed systemic inflammatory disorders, increased effector T cells, and reduced Treg cell function. Heterozygous RhoA deletion led to Treg cell plasticity without autoimmunity and suppressed tumor growth by enhancing anti-tumor T cell immunity. Overall, RhoA maintains Treg cell balance and restrains autoimmune responses, while its deficiency enhances Treg cell plasticity with implications for autoimmune diseases and anti-tumor immunity.

Sanders et al. (**Table 1**) focused on Foxp3^+^ regulatory T cell therapy for tolerance. Solid organ transplantation (SOT) is vital for treating end-stage organ disease, but chronic rejection poses challenges due to lifelong immunosuppressive drug use. Autoimmune diseases also disrupt self-tolerance, affecting patient health and healthcare costs. Tregs play a key role in maintaining self-tolerance. Understanding Treg function offers hope for new therapies in both SOT and autoimmune diseases. Dysregulation of Tregs is observed in autoimmune diseases, leading to higher disease activity. In transplantation, Tregs promote graft tolerance and prevent rejection by suppressing alloreactive T cells. Adoptive cell transfer techniques, including chimeric antigen receptor (CAR) Tregs, show promise in both contexts. Ongoing research aims to optimize Treg-based therapies for clinical use. Challenges include ensuring Treg specificity and long-term viability. Despite hurdles, Treg-based therapies offer significant potential for treating autoimmune diseases and improving transplant outcomes.

The current topic regarding the role of T helper cells in autoimmune diseases has attracted timely reviews, studies based on samples from AD patients, and a mechanistic study in a mouse model. We hope it will contribute to understanding the role of T cells in these devastating diseases that present with a complex biology.

## Author contributions

DH: Writing – original draft, Writing – review & editing. VO: Writing – original draft, Writing – review & editing. PH: Writing – original draft, Writing – review & editing.
